# Tumor of Hypophysis

**Published:** 1913-11

**Authors:** 


Tumor of Hypophysis. Rozabel, *Revista de Med. y Cir. Pract.*, September, 1912, presents two cases in brothers. Parents normal except for short stature and repeated abortions, Wasserman reaction negative in both. The brothers, at fourteen and eleven, showed increased fatty tissue, stunted growth, infantile genitals, double optic atrophy, amaurosis in older, hemianopsia in younger child. Both had a supernumerary digit on each foot and the younger had a hypospadias. X-ray showed no abnormality of the sella turcica.

